# Statistical Guidance for Experimental Design and Data Analysis of Mutation Detection in Rare Monogenic Mendelian Diseases by Exome Sequencing

**DOI:** 10.1371/journal.pone.0031358

**Published:** 2012-02-10

**Authors:** Degui Zhi, Rui Chen

**Affiliations:** 1 Section on Statistical Genetics, Department of Biostatistics, University of Alabama at Birmingham, Birmingham, Alabama, United States of America; 2 Department of Molecular and Human Genetics, Baylor College of Medicine, Houston, Texas, United States of America; Charité Universitätsmedizin Berlin, NeuroCure Clinical Research Center, Germany

## Abstract

Recently, whole-genome sequencing, especially exome sequencing, has successfully led to the identification of causal mutations for rare monogenic Mendelian diseases. However, it is unclear whether this approach can be generalized and effectively applied to other Mendelian diseases with high locus heterogeneity. Moreover, the current exome sequencing approach has limitations such as false positive and false negative rates of mutation detection due to sequencing errors and other artifacts, but the impact of these limitations on experimental design has not been systematically analyzed. To address these questions, we present a statistical modeling framework to calculate the power, the probability of identifying truly disease-causing genes, under various inheritance models and experimental conditions, providing guidance for both proper experimental design and data analysis. Based on our model, we found that the exome sequencing approach is well-powered for mutation detection in recessive, but not dominant, Mendelian diseases with high locus heterogeneity. A disease gene responsible for as low as 5% of the disease population can be readily identified by sequencing just 200 unrelated patients. Based on these results, for identifying rare Mendelian disease genes, we propose that a viable approach is to combine, sequence, and analyze patients with the same disease together, leveraging the statistical framework presented in this work.

## Introduction

One of the major gaps in our understanding of human genetic diseases is to fully categorize their molecular basis. To date, the underlying mutations for at least 3000 human disease loci remain to be determined (http://www.ncbi.nlm.nih.gov/Omim/mimstats.html). Recent developments in high throughput sequencing technologies provide an opportunity for accelerating the disease gene identification process. In the past two years exome sequencing, an economical alternative approach to whole genome sequencing, has achieved ground-breaking success in identifying genes associated with rare monogenic Mendelian diseases (RMMDs). In these studies, a number of unrelated patients with the same rare genetic disease were exome-sequenced to identify coding variants [1], [2].

Ng et al. [2] were the first to demonstrate the effectiveness of this approach: by sequencing the exomes of four patients of the Miller syndrome from three kindreds, they identified the gene *DHODH* as the sole candidate. Subsequently, exome sequencing has been successfully applied to several rare Mendelian disorders with a monogenic component [3], [4], [5], [6], [7], [8], [9], [10], [11], [12], [13]. While most of these cases were recessive diseases[1], [2], [4], [5], [6], [7], [8], [9], [11], [12], [13], this approach can be also applied to dominant diseases [3], [10], albeit substantially more complex bioinformatics analyses are required. Family history data are extremely helpful as they can narrow down the scope of search for disease mutations from genome-wide to co-segregation or identical-by-descent regions [4], [5], [6], [7], [10], [11], [12], [14], [15]. While exome sequencing has been widely used due to its relatively low cost and clear interpretation of identified changes, whole genome sequencing has been used to identify both coding and non-coding mutations [14], [15], [16].

It is noted [17] that the exome sequencing for rare monogenic Mendelian diseases (exome-RMMD) and that of exome sequencing for complex traits (exome-GWAS) are two distinctive experimental designs that applied to diseases with different genetic architectures: While exome-RMMD assumes that rare Mendelian diseases are caused by rare genetic variants with complete or very high penetrance, exome-GWAS design does not assume that complex traits are caused by rare variants nor complete penetrance. As a result, exome-RMMD and exome-GWAS engage largely different analysis approaches [17]. There have been enthusiasms and preliminary studies regarding exome-GWAS, and some works [18], [19] exist for the statistical guidance and power considerations for exome-GWAS, there has been a lack of statistical framework for exome-RMMD. This work is focused only on Mendelian diseases or complex diseases that transmit in a Mendelian fashion in families. Notably the exome-RMMD design may apply to a disease cohort with a large number of unrelated individuals (e.g., n = 500–1000) with a high degree of locus heterogeneity, which resembles classical whole-genome association studies for complex traits. The main difference of exome-RMMD and exome-GWAS is not in the size of the cohort but rather on the underlying genetic architecture, although exome-RMMD typically requires a smaller sample size.

Despite these successes on exome sequencing approach for rare mendelian diseases, concerns remain regarding the feasibility of extending this approach to Mendelian diseases more broadly. First, there is a concern on publication bias. Some successful publications on using very small number of families to find causative genes for Mendelian diseases may not indicate that all rare Mendelien diseases can be solved this way. We are aware that many studies suffer from uncertainty and limited power to trim down the final candidate gene list. Therefore, a statistical modeling framework is needed for the guidance for study design and data analysis.

Specifically, there is a concern on factors that can negatively impact the utility of this approach. First, limitations of exome capture sequencing technology result in both false negatives and false positives in mutation detection. In the case of false negatives, pathogenic variants may not be detected in any given sample due to insufficient sequence coverage of some exonic and non-exonic regions. In contrast, false positives resulting from both short read mapping and sequencing errors are commonly observed with current sequencing technologies. Second, distinguishing causative mutations from other non-causative rare variants in patients is often not straightforward. Each individual harbors hundreds of rare and private variants [20]. Our ability to predict the functional significance of these rare variants is still very limited. Third, most Mendelian diseases are highly heterogeneous at both the clinical and the molecular levels. Most genetic diseases are locus-heterogeneous as mutations in any one of many genes can cause similar clinical phenotypes. Often mutations in a single gene account for only a small portion of the patients (<10%). In such cases, simply intersecting candidate genes derived from sequencing of several patients is unlikely to lead to identification of disease genes. It is essential, therefore, to systematically evaluate the impact of these factors on the statistical power of disease gene identification by exome sequencing in order to evaluate and guide experimental design.

In this report, we present a formal analytical framework for exome sequencing studies for RMMDs. Our framework establishes a quantitative link between the statistical power and various disease and experimental variables. Based on our model, we found that underlying mutations and genes can be reliably identified by sequencing a moderate number of patients for recessive RMMDs with substantial locus heterogeneity. In contrast, a greater number of patients or additional genetic mapping data is needed for mapping genes of dominant RMMDs. Validated by computer simulations and real data, a web analytic tool has been implemented which can be used as a guide for both experimental design and data analysis. Based on our results, we confirmed that a viable approach for identifying RMMD disease genes is to combine patients with the same clinical disease and to perform exome sequencing and subsequent analysis together. Moreover, this approach may be applicable to disease cohorts with extensive genetic heterogeneity, leveraging the statistical framework presented in this work.

## Results

### Modeling framework

As listed in Table 1, we identified a list of relevant experimental and disease factors which are likely to impact experimental results. A typical exome-sequencing study for a rare Mendelian disease consists of a number of unrelated patients (denoted by *n*). DNA samples of these individuals are subjected to exome capture and sequencing. The number of genes (denoted by *M*) covered by the exome capture procedure varies depending on the capture design. Obviously causative genes missed by the capture procedure cannot be identified by this approach and account for upfront power loss regardless of downstream filtering and statistical analysis. Our framework is thus purely focused on the statistical power within the captured region and the overall power of the exome sequencing experiment should be actually smaller. For each sample, a preliminary list of putative variants identified by sequencing will be subject to filtering procedures such as excluding common variants in the human population, low quality variants, and synonymous changes, resulting in *m* candidate mutations. In cases where genetic mapping information is available, variants mapped outside of disease loci identified by homozygosity mapping [5], [6] or linkage analyses [15] can also be excluded, resulting in a shorter list of candidate mutations. These *m* variants would include zero or one disease-causing mutation(s) while the rest are rare or private non-causative mutations and thus serve as a measure of level of false positives. The probability that a disease-causing mutation is present in the final list of *m* variants is denoted as the sensitivity of mutation detection, *Ps*. While the present work focuses on the statistical power assuming the *m* and *Ps* are given, an important decision faced by investigators is to choose a proper filtering procedure: a more stringent filtering would reduce false positive (smaller *m*), but at the same time reducing the power of detecting the true disease-causing mutations (smaller *Ps*). The complex relationship between *m* and *Ps*, depending on the details of filtering and the nature of the disease, is out of the scope of the present work.

**Table 1:** Experimental design and disease factors of the causative gene relevant to the statistical power of exome sequencing for RMMDs.

Factor	Symbol	Type	Definition	Impact to power when other factors held constant
Sample size	*n*	Design	Number of unrelated patients sequenced	increase
Locus heterogeneity	*R*	Disease	Proportion of sequenced patients responsible	decrease
Dominant/Recessive		Disease	Genetic link of gene to disease. Dominant = 1, recessive = 0	decrease
Relative gene length	*w*	Disease	Ratio of the length of the gene to the average gene length	decrease
Sensitivity of detecting mutations	*Ps*	Design	Probability of a true mutation in the captured region being identified after filtering	increase
Filtering efficiency	*m*	Design	Number of mutations identified after filtering	decrease
Genome size	*M*	Design	Total number of genes in the captured region	decrease

While all these factors (*n*,*M*,*m*,*Ps*) are affected by experimental design, execution, and subsequent data analysis, other factors that are intrinsic to the underlying disease also need to be considered. Three intrinsic factors have been identified, including the mode of inheritance (dominant or recessive), the fraction of sequenced patients for which a given gene is responsible (denoted by *R*), and the conditional probability that a random mutation falls in the gene, given that there is a mutation. The latter is proportional to the gene length, referring to the total lengths of the exons of a gene, and the background mutation rate in the gene region. For the sake of simplicity, we use the relative gene length (denoted by *w*), defined as the ratio between the candidate gene size to the average gene size in the genome, to incorporate the probability of having a random mutation in the gene, recognizing that a complete treatment would also incorporate the background mutation rate information.

As will be detailed in the Materials and Methods section, we examined three test statistics at the gene-level, including *Ta*, *Tr*, and *Td*. For a gene, the basic test statistic is the total variant count among all sequenced patients. This statistic is denoted as *Ta* since it is extended from an additive model. For a recessive model, Ng et al [2] used the filter requiring at least two mutations in the gene. This motivates us to define the recessive version of the statistic, *Tr*, as the count of patients with at least two mutations in the gene. Analogously, we denote the count of patients with any number of mutations in a gene, or the collapsed count, as *Td*, the dominant version of the statistic. We assume that mutations occur in a gene randomly with a probability proportional to *w*, the relative gene length. We further assume that different mutations occur independently, i.e., there is no linkage disequilibrium between these rare mutations. It can be derived, with a tight approximation, that each of these statistics *Ta*, *Tr*, and *Td* follows a different binomial distribution under the null hypothesis where there is no association between the gene and the disease. The parameters of the binomial distribution are determined by *n*, *w*, *m*, and *M*. Based on these binomial distributions, it is appropriate to conduct exact binomial tests and the type-I error rate and significance-level cutoff can be determined. The p-values are subject to Bonferroni correction controlling for the fact that a total of *M* hypotheses, one for each gene, are being tested genome-wide. Upon multi-testing correction, results obtained from theoretical calculations are consistent with those obtained from computer simulations (Table S1).

As will be detailed described in the Materials and Methods section, given the null distribution, the power of a binomial test can be derived for all three statistics, *Ta*, *Tr*, and *Td*. Our derivations are based on the following realistic assumptions. First, we assume that in the recessive case exactly two causal mutations after filtering per individual are present in the gene. This is plausible as individuals with homozygous or compound heterozygous mutations would incur severe damage to their fitness and thus unlikely to produce offsprings with additional mutations. Similarly, we assume that in the dominant case exactly one causal mutation after filtering is present in one copy of a gene per individual. Based on these assumptions, it can be derived that each of the statistics *Ta*, *Tr* and *Td*, follow a different binomial distribution, with a higher mean than the null distribution, except that under the recessive model *Ta* follows a distribution closely resembling binomial. Based on this analytical framework, the effect of all factors listed in Table 1 on the experiment can be systematically evaluated by theoretical power calculations, with all calculated results being validated by computer simulations (data not shown).

We remark that all proposed test statistics, *Ta*, *Tr* and *Td* are different from many test statistics proposed from rare variant association (as reviewed by several papers including [17], [21], [22]). Primarily, the proposed methods are “case-only” statistics since exome-RMMD is a case-only design and individuals' phenotypes are largely ignored, this is fundamentally different from rare variant association methods whose very goal is to identify the statistical association between individuals' genotypes and phenotypes. For example, even though the Ta statistic resembles the simple sum test statistic [23] and the Td statistic resembles the collapsing method [24], these rare variant association methods only combine the information across multiple variants in a region for an individual, while the proposed methods further collapse individual-level statistics into a single statistic.

### All factors directly impact the power of mutation detection

**Figure 1:**
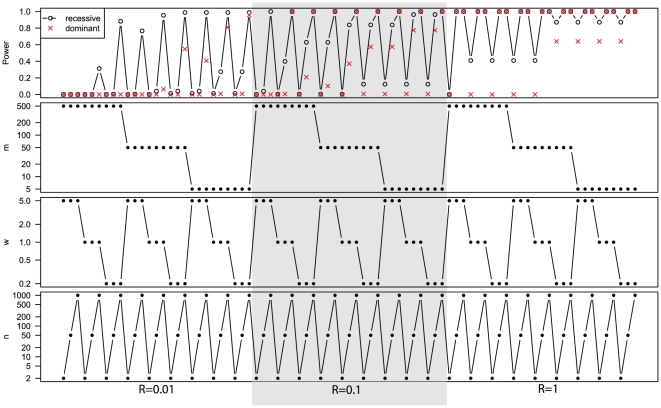
The calculated power of exome sequencing for rare monogenic Mendelian diseases for various parameter combinations.

The statistical power of exome sequencing for rare monogenic Mendelian disease is high for genes with a recessive link (versus a dominant link) to the underlying disease, with a low genetic heterogeneity (1/*R*), or of a short length (*w*) (Figure 1). Moreover, high filtering efficiency (1/*m*), large sample size (*n*), and high sensitivity of mutation detection (*Ps*, data not shown) can boost the power further. In a near optimal combination, *R* = 1, *Ps* = 0.9, and *m* = 5, even a sample size of two almost guarantees identification of the gene. Below we discuss in detail the effect of individual parameters while fixing the remaining parameters to the following default values: *R* = 0.05, *w* = 1, *Ps* = 0.8, *m* = 300, and *M* = 20,000. The justifications for these choices are given below in the discussion of individual factors.

### Genes underlying highly heterogeneous diseases can be identified by sequencing a moderate number of patients

Most Mendelian diseases are genetically heterogeneous and quite often mutations in one gene account for only a small fraction of patients in a sample collection. To evaluate the impact of heterogeneity on the power to identify disease genes, we vary the fraction of cases caused by mutation in the same gene, *R*, from 0.01 to 1.

**Figure 2:**
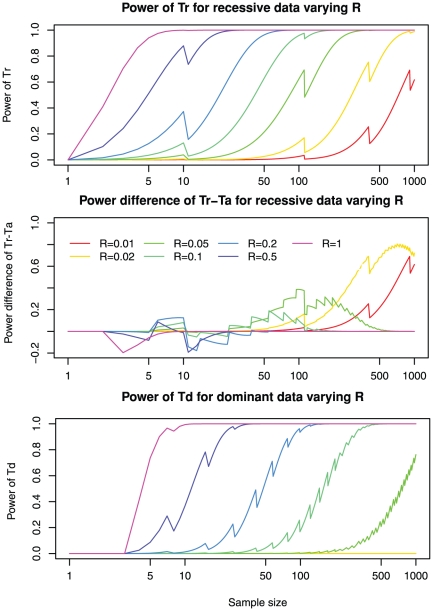
Genes underlying highly heterogeneous diseases can be identified by sequencing a moderate sized sample. The calculated power with varying degrees of genetic heterogeneities (*R*) ranging from 0.01 to 1 is shown. Upper panel: power of *Tr* for detecting a recessive gene; Middle panel: power difference *Tr*-*Ta* for detecting a recessive gene; Lower panel: power of *Td* for detecting a dominant gene. Other parameters are fixed to the default values: number of mutations *m* = 300; total number of genes *M* = 20,000; sensitivity of detecting mutations *Ps* = 0.8; and the mutation probability equals genome-wide average *w* = 1. See Tables S2, S3 and S4 for more dense sampling of *R* values. Note that power does not always increase monotonously with sample sizes (zigzag line patterns). The loss of power upon increase of sample size is related to discrete changes in the significance level cutoff 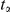 of the test and thus very small test size (not close to 0.05) as shown in Table S1, since the distribution of the test statistic is discrete.

Under a recessive model, power is high with either large sample sizes or low genetic heterogeneities (Figure 2, upper panel). For example, when *R* = 1, just two patients will already render a power of 0.41 for *Tr*. When *R* = 0.2, power of *Tr* is high (>0.8) for *n* = 40. At very low *R* values, e.g., *R* = 0.05, sample size must be large (*n* = 200) to achieve sufficient power. At the extremely low values of *R* (e.g., 0.01), one would not expect to map a gene with *n* = 100 samples, because the expected value of *Tr* equals one. However it is remarkable that *n* = 1000 is sufficient to maintain a modest power (>0.5).

The sensitivity of detecting causative mutations by exome sequencing, *Ps*, is also an important factor. When taking *Ps* to the default value of 0.8, in some cases (Figure 2, middle panel), the additive version of the T-statistic *Ta*, instead of the recessive version of the T-statistic *Tr*, can be used to identify genes that are enriched for rare changes. For large *R* values (i.e., *R*≥0.1) at small sample sizes, *Ta* actually can have a higher power than *Tr*. *Tr* is more powerful for *R*≤0.1. The reason for this counterintuitive behavior of *Tr* and *Ta* is the following: Imperfect sensitivity of mutation detection (e.g., *Ps* = 0.8) always results in some power loss. For a causal gene with two true mutations, there is a chance that only one mutation is not detected in an individual. While *Tr* will lose one count from this individual, *Ta* can still collect one count from this individual and thus the loss of power for *Ta* might be smaller. Indeed, when sensitivity of detecting mutations is perfect (*Ps* = 1), *Tr* is universally more powerful than *Ta*. In any case, *Tr* universally has better power to detect mutation than *Td* for recessive data (Figure S1).

Many rare diseases are dominantly inherited. In this case, the criteria for calling a gene positive in an individual are different from that of recessive diseases, and the power for identifying causative genes in dominant diseases is substantially lower than that of recessive diseases. The power of detecting dominant disease genes at various *R* levels is calculated and shown in Figure 2, lower panel. Under a dominant model, power can be good for modest sample sizes, when *R* is sufficiently large. For example, when *R* = 0.5, power is good (>0.8) for *n* = 20. When *R* = 0.2, power is good (>0.8) for *n* = 70. At very low *R* values, e.g., *R* = 0.05, even a very large sample size (e.g., *n* = 1000) can only offer a power of 0.76. When *R*<0.05, no sample size smaller than 1000 would be sufficient.

### High sensitivity of detecting mutations is required to identify disease genes

**Figure 3:**
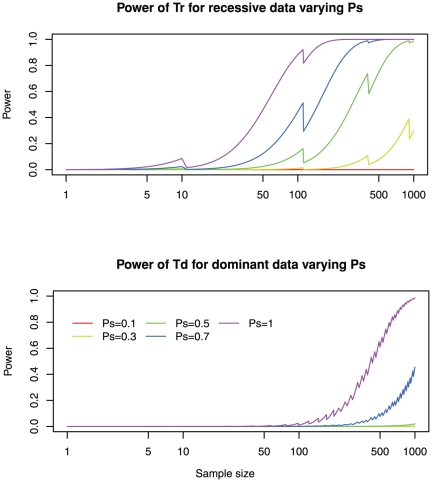
High sensitivity of detecting mutations is required to achieve a useful power. The power for varying degrees of sensitivities of mutation detection, ranging from 0.1 to 1 is shown. Other parameters are fixed to the default values: number of mutations *m* = 300; total number of genes *M* = 20,000; genetic heterogeneity *R* = 0.05; and the mutation probability equals genome-wide average *w* = 1. See Tables S5 and S6 for more dense coverage of sensitivities of mutation detection.

Sensitivity of detecting mutations in an individual is mainly affected by three factors: the coverage of the capture technology, the sequencing quality, and the read mapping quality. Various capture methods have been developed to enrich the coverage of human exons. Unfortunately, none of the current methods can capture all exons and typically 10–15% of exons remaining poorly covered. Since the ceiling of coverage is often beyond investigators' control, we define the sensitivity of detecting mutations *Ps* as the probability of detecting a mutation within the scope of exon capture technology. Fortunately, with the advancements of next-generation and possibly third-generation sequencing technologies, higher sequence coverage and low sequencing error rates can be achieved at an affordable cost. For heterozygous sites, it has been estimated that about 20× coverage is required to reliably detect both alleles. Still, it is well known that the sequencing coverage and read mappability is not uniform across the genome and thus *Ps* would fluctuate from gene to gene and within a gene. Mutations at certain nucleotide positions may be difficult to detect for any patient sequenced. Therefore, although an overall 97% sequencing coverage of the captured region is reported with current technology [2], we take a somewhat conservative value *Ps* = 0.8 in our discussion. Sufficient sensitivity of detecting mutations (*Ps*>0.7) is required to achieve an adequate power even when sample size is large (Figure 3). In practice, maintaining *Ps*≥0.9 is reasonably affordable and is sufficient to attain the desired power. Moreover, extremely high coverage does not yield a good return on investment.

### Strict filtering of false positives has limited impact on mapping recessive disease genes but can dramatically improve the power of mapping dominant disease genes

The advantage of exome sequencing for RMMDs is that, based on the assumption that the disease is caused by rare variants, common variants can be safely filtered out using existing SNP databases, and typically only about a few hundred mutations (*m*) would remain in an individual. For reference, counting new (not in dbSNP129, 1000 genomes, nor control exomes) NS/SS/I (nonsynonymous, splice site, and short coding indel) variants per patient, Ng et al identified *m* = 526 in their four patients Miller syndrome sample [2], and about *m* = 694 (Table 1 of Ng, et al. [3]) in their 10 patients Kabuki syndrome sample [3]. Using a more strict loss-of-function filter, they identified *m* = 75 for the latter study [3]. Noting that m can be reduced further if additional linkage mapping information is considered, we set the default value *m* = 300 and evaluate the statistical power for detecting disease genes for the range from *m* = 5 to *m* = 500.

**Figure 4:**
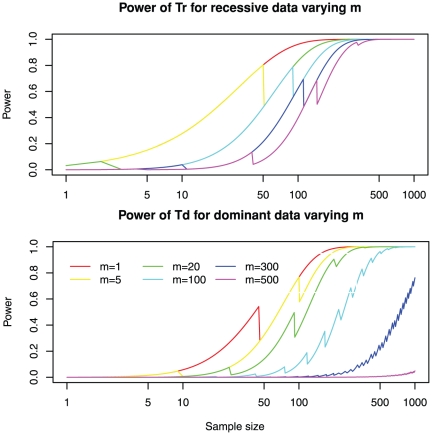
Strict filtering of false positives has limited impact on recessive diseases but dramatically reduces the power of detecting dominant disease genes. The power for varying degrees of filtering efficiencies, ranging from 5 to 500, is shown. Upper panel: power of *Tr* for recessive data; Lower panel: power of *Td* for dominant data. Other parameters are fixed to the default values: genetic heterogeneity *R* = 0.05; total number of genes *M* = 20,000; sensitivity of detecting mutations *Ps* = 0.8; and the mutation probability equals genome-wide average *w* = 1. See Tables S7 and S8 for more dense coverage of filtering efficiencies.

Here we analyze the effect of these filters on the power of exome sequencing for RMMDs. As expected, higher filtering efficiency (smaller *m*) increases the power (Figure 4). Interestingly, filtering efficiency has a more dramatic effect for dominant models than for recessive models. For example, reducing *m* from 500 to 50 for *n* = 200 can only improve power from 0.769 to 0.989 for a recessive model, but can improve power from 0 to 0.692 for a dominant model.

It is worth noting that when *m* is small (*m*<30), there is some power for the recessive model even for a single patient *n* = 1. This result can be more dramatic if *R* is larger than the default of 0.05. In fact, when *R* = 1 and *m* = 5, the power for a recessive model for *n* = 1 is 0.64.

There are three main strategies to reduce *m*. First, *m* can be reduced by combining linkage information. Second, *m* can be reduced by more fine-tuning of SNP filtering, fueled by the development of public SNP databases. Finally, *m* can be reduced further by applying SNP functional annotations. For example, loss-of-function filters that select only premature stop-codons and frameshifts have been productive (Ng et al [3]). Moreover, functional prediction of variants provided by programs such SIFT [25], PolyPhen [26], and the genomic evolutionary rate profiling (GERP) score [27] can be applied. However, these function predictions are not yet sufficiently accurate (Ng et al [2]; Ng et al [3]). Finally, function prediction filtering is a double-edged sword: while it eliminates false positive by reducing *m*, it can also filter out true disease-causing mutations (reducing *Ps*) and thus hurt the statistical power.

### Power is low for long genes

In the calculations above we assume that the probability of a random mutation falling in a gene is equal to the genomic average 1/*M*. In reality the probability of a random mutation falling in a gene may fluctuate depending on the gene size and the local mutation rate. For convenience, we will interpret *w* of a gene as the total length of its exons.

**Figure 5:**
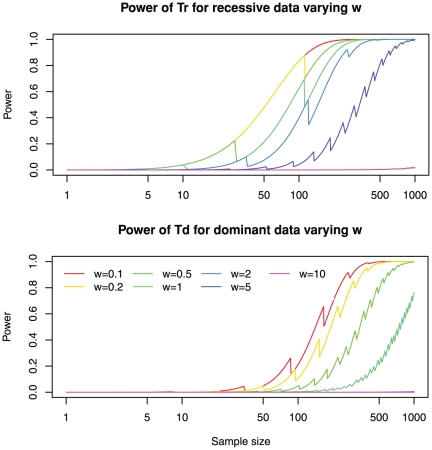
Power is low for long genes. The power for varying degrees of relative mutation probabilities, ranging from 0.1 to 10 times the genome average is shown. Upper panel: power of *Tr* for recessive data; Lower panel: power of *Tr* for recessive data. Other parameters are fixed to the default values: number of mutations *m* = 300; genetic heterogeneity *R* = 0.05; total number of genes *M* = 20,000; and sensitivity of detecting mutations *Ps* = 0.8. See Tables S9 and S10 for more dense coverage of filtering efficiencies.

Gene size has a strong influence on power (Figure 5). Power for long genes is low: for a recessive model with *w* = 10 or a dominant model with *w*≥2, there is nearly no power at all. On the other hand, there is a limited gain in power for shorter genes: there is not much difference between *w* = 0.1 and *w* = 0.2 for both recessive and dominant models. In practice, it should be critical to include gene size for calculating P-values, as illustrated below.

### Re-analyses of published data

We tested whether our framework can help guide both experimental design and data analysis in recently published exome sequencing studies [2], [3]. Exome sequencing was conducted to investigate Miller syndrome, a recessive disorder, for four patients from three kindreds (Ng, et al. [2]). The relevant parameters were *m* = 526, *M* = 17,000, *Ps* = 0.97, and *n* = 3. This is a sufficiently powered design: the retrospective power calculated with these parameters would be 0.99 (using the *Tr* statistic) for discovering a gene of average length and no locus heterogeneity (*R* = 1). Using the actual data from this study, we estimate that *w* would be 0.736 as the average length of proteins in the CCDS 2008 (20090327), the target exome capture set, is 538 aa, and the identified gene *DHODH* encodes a 396 aa protein. As a result, the calculated p-value would be 3.2×10^−6^, more significant than the p-value of 1.5×10^−5^ reported (Ng, et al. [2]). Therefore, analysis under our framework is consistent the study design and the data analysis of Ng, et al. [2], but gives more quantitative details.

Exome sequencing is less-powered for dominant diseases. As indicated from the model, lowering the level of false positives (small *m*) is the key for identifying mutations underlying dominant diseases. This is consistent with data presented in a study in which exome sequencing was conducted for 10 unrelated individuals with Kabuki syndrome, a dominant disorder (Ng et al [3]). After allele frequency based filtering using dbSNP, 1000 Genome projects, and control exomes, an average of 694 candidate genes per patient were identified (Table 1 of Ng, et al. [3]). They found that seven out of the 10 patients carry rare variants in the *MLL2* gene. However, since *MLL2* is about ten times the average gene size, this observation (*Td* = 7) is actually not statistically significant (p = 0.007 before Bonferroni correction). When a stringent loss-of-function filter was applied, the false positive rate is reduced by nine fold with each individual having an average of 75 mutations. As a result, the p-value for observing seven out 10 patients carrying rare variants in *MLL2* become 6.98×10^−5^ and is statistical significant. Interestingly, *MUC16* was considered as a “likely false positive due to its extremely large size” although all 10 patients carried rare mutations in this gene (Ng, et al. [3]). Indeed our analysis confirmed this claim: due to the large coding region size (14,507 aa), the p-value for finding mutations in *MUC16* in 10 out of 10 patients is still not significant even before Bonferroni correction. In other words, using p-values of the *Td* statistic, *MUC16* should be ranked lower than *MLL2* even though more individuals carry *MUC16* mutations.

The data analysis of exome sequencing experiment can be more challenging than merely filtering of variants using existing SNP databases. In fact, Ng, et al. [3] developed a *post hoc* ranking scheme for candidate genes. They first assign case scores to patients based on their phenotypes and functional prediction scores to individual variants, and then rank the candidate genes by the summation of case scores and variant scores. A more rigorous analysis of exome sequencing data should be under a formal statistical framework, and our work provides a start toward this direction.

## Discussion

Exome and whole genome sequencing of patients are becoming a major approach for unlocking the molecular basis of uncharacterized human rare Mendelian disease loci. In this report, we have identified various disease and design factors that influence the statistical power of this approach. An analytical framework that quantitatively links these factors to statistical power has been established. This model is validated by computational simulation. As expected, the statistical power of identifying disease genes is affected by both experimental conditions as well as intrinsic features of the diseases. Importantly, based on our model, for recessive Mendelian diseases, the vast majority of disease genes can be readily identified when a moderate number of patients with the same disease are sequenced and analyzed together. This is true even when the heterogeneity of the disease is high. For example, in the case of recessive disease, a power of 0.89 can be reached for identifying a gene responsible for as little as 5% of the disease population by sequencing 200 unrelated patients. In contrast, the power for dominant diseases is substantially lower where sequencing of more than 1000 patients is needed to achieve a comparable power. Our result is significant since it indicates that the molecular basis for the vast majority of uncharacterized recessive disease loci can be resolved using the exome sequencing approach.

Our framework can provide guidance for both experimental design and data analysis. In general, proper combination of sufficient sample size, capture sequencing coverage, cutoff for variants identification, stringency of variants filtering, and inclusion of genetic mapping information are important to maximize the success of exome sequencing experiments. However, strategies used to tackle recessive versus dominant disease are quite different. In the case of recessive disease, the key factor is the sample size. Based on our model, genes underlying highly heterogeneous recessive diseases can be identified by sequencing a moderate number of patients. In contrast, since the *Tr* statistic, counting the number of individuals with ≥2 mutations is already quite effective for recessive diseases, reducing false positive mutations by aggressive allele frequency filters and bioinformatic filtering have only a minor impact on improving power. In the case of dominant diseases, the key factor is to reduce the number of candidate variants. Both aggressive filters and genetic mapping should be implemented to maximize the exclusion of variants in order to improve the power. In contrast, although positively correlated, increasing sample size has limited impact on the power for highly heterogeneous dominant diseases. Other than variant filtering and sample size, a common factor important for experimental design is the underlying heterogeneity of the disease. To increase power, it is highly desirable to minimize heterogeneity. This may be achieved by grouping patients based on their clinical phenotype. In addition, reiterating the analysis by excluding samples with already identified causative mutations can also be informative. An often overlooked but potentially confounding factor to be considered during data analysis is the length of the gene. As genes with large size incur more rare variants by chance, it is important to adjust the statistical significance of findings based on gene size. To facilitate the ranking of putative disease genes, the binomial test p-values proposed in our report can be calculated for each candidate gene, which provides a unified metric to rank genes similarly to what is used in Genome-wide Association Studies (GWAS) analyses. To facilitate experimental design, statistical power estimation, and p-value calculation, an online calculator has been developed and can be accessed at http://exomepower.ssg.uab.edu.

Our results show that, for rare monogenic Mendelian diseases, it would be feasible to apply the exome-sequencing approach to discover causative genes even when a substantial level of genetic heterogeneity exists among patients. This can be achieved by conducting rigorous statistical tests that can evaluate the statistical significance of identified mutations present in a small portion of a relatively large collection. Therefore, a key to identifying genetically heterogeneous rare Mendelian disease genes is to collect large samples of patients and analyze the sequence data together. As patients with mutations in individual disease genes are rare, it will be more efficient and powerful to combine samples with the same disease from multiple collections for sequencing. In effect, the study of rare disease is not unlike the study of common diseases in which investigators form large consortiums to achieve a sufficiently powered sample size. Given a sufficient number of samples, the lack of extended family data, a major bottleneck for linkage-based disease gene mapping approaches, does not pose a substantial problem for exome sequencing.

Admittedly, in this work we adopted a simple statistical framework. Real RMMD exome data analyses often involve in applications of a number of filters. There are several directions where a more advanced statistical framework could be established. First, the current framework assumes there is only a single mutation filter. In real data analysis there is often an array of filters, each with a different set of criteria, that are applied in combination. It is an interesting question how to best combine these filters and adjust the p-values accordingly. Second, the current framework adopts simple mutation count statistics. It may be useful to take into account the strengths of different types of mutations and the phenotypic differences among patients, such as the weighted sum statistics [28] and the post hoc score developed by Ng et al [3]. Third, explicit modeling of disease heterogeneity, either phenotypic or genetic, should be explored as well. Fourth, the proposed test for recessive diseases simply requires that at least two mutations are present in a same gene, as haplotype information of these mutations are typically unavailable. It is possible, with improved genotype and haplotype calling algorithms or longer sequencing reads, that haplotype information can be estimated or observed, and thus one can improve the recessive test by requiring two mutations to be on different chromosomes. Fifth, mutation filters may be applied based on allele frequencies. Our discussion was mostly focused on strict filters which assume that disease-causing mutations are not present in any of healthy individuals. While this is likely true for dominant diseases and very rare recessive diseases, it may not be true for rare recessive diseases with a moderate prevalence, in which case mutations may be present in healthy individuals in heterozygous state. In that case, filters based on a certain allele frequency cutoff may be more appropriate. Sixth, software tools predicting variants' pathogenicity such as PolyPhen2 [26], SIFT [25], and MutationTaster [29] are often used. The statistical properties of these filters may be studies in future research. Seventh, while this work is primarily focused on exome sequencing, the main results are also applicable to the analysis of the genic portion of whole genome sequencing for rare diseases [15]. Finally, many successful discoveries of disease-causing genes of RMMD by exome sequencing capitalize on the rich information on family information. For example, rare recessive diseases often run in highly inbred families in which patients often carry a common homozygous mutation. While our model is designed for exome sequencing of unrelated individuals of rare Mendelian diseases, it offers insights into two factors that may explain the high rate of success of familial exome sequencing: This would be a special case with zero genetic heterogeneity (*R* = 1). Also, very strict filtering criteria requiring disease causing mutations to be homozygous can be used, resulting very small *m*.

## Materials and Methods

### Setup of the framework

An exome-sequencing study for a rare disease consists of *n* unrelated patients. Suppose *m* mutations survive rigorous filtering, scattered among a total of *M* candidate genes. For simplicity we assume that the number of surviving mutations is the same for each individual sequenced patients. In practice the number will vary between individuals but the variation is likely small. The raw data collected is an *n*-by-*M* count matrix, *C*, in which element *C_ij_* is the number of mutations at gene *j* for individual *i*. *X_ij_* is the coding of genotype at gene *j* for individual *i*. Under a recessive model, 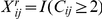, where *I*(*x*) is the indicator function taking 1 if *x* is true, and 0 otherwise, and the superscript *r* denotes the recessive model. Under the dominant model, 
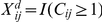
, and the superscript *d* denotes the dominant model. There is no additive model for Mendelian diseases, but for the sake of completeness, the genotype coding for an additive model is 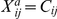. As in most association studies, we are interested in the statistic 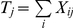 for gene *j*, as it aggregates information across multiple patients. There are three versions of the T-statistics, *Tr*, *Td*, and *Ta*, for recessive, dominant, and additive models, respectively.

### Type-I error rate and significance-level cutoff

We focus our discussion on single gene based test and consider one gene of interest, namely, gene *j*, at a time. Under the null hypothesis, gene *j* is not associated with the disease, and all *m* mutations identified after filtering are random non-causal mutations. We first assume that gene *j* is of average length and the conditional probability of each of the *m* mutations landing on gene *j*, given that there is a mutation, is 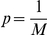. This is obviously simplistic and we will provide treatment for different gene length in later discussion. As a result, the mutation count of gene *j*, given that there are total *m* mutations, follows a binomial distribution: 
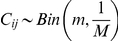
. We remark that this is not a hypergeometric distribution as mutations can land on the same gene multiple times. Since we only focus on a single gene at a time and omit the subscript *j* in the following discussions. For a typical exome sequencing project for a Mendelian disease after rigorous filtering, *m* is much smaller than *M* and thus 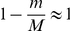. It can be shown that 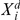 for a dominant model is a Bernoulli random variable with 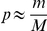, and 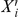 for a recessive model is a Bernoulli random variable with 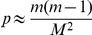 and these approximations are very tight (see Document S1). Therefore, the dominant-version of the T-statistics is
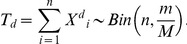



Similarly, the recessive-version of the T-statistics follows
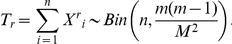



Moreover, the additive-version of the T-statistics is
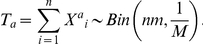
.

In reality the probability of a random mutation falling on a gene may fluctuate depending on its size and its local background mutation rate. Assume a gene with a probability *p* that is *w* times of the genomic average to carry a mutation, i.e., 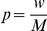, we can use 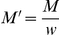 and the above derivations still apply if we substitute *M* with *M′*.

Based on the above derivations, an exact binomial test can be implemented where the score cutoff 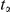 of a test statistic *T* for a given significance level 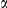 is set to be 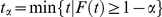, where 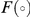 is the binomial cumulative distribution function of *T*. Since we are considering a total of *M* potential hypotheses, one for each gene, a multiple testing correction is required. We adopt the Bonferroni correction in the present work, where the significance level is set to be 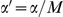. Notice that the binomial distribution is discrete and thus for a fixed 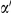, the cutoff 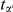 can be a stepwise function of the sample size *n*. This explains the non-continuous nature of the power curves in the Figures 2, 3, 4, and 5.

### Power calculation

For a gene with a recessive link, for a patient, we assume that there are exactly two mutations, one on each chromosome, in the gene of interest. There is a probability *R* that the gene carries these two mutations. When the gene carries the two mutations, there is a probability *P_s_* to discover either of them. Therefore, the distribution of the number of mutations under a recessive model, 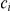, would be a “trinomial” distribution:
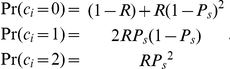



The distribution of the recessive version of the T-statistic for a recessive gene is
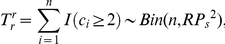
where the superscript *r* stands for recessive genetic model and the subscript *r* stands for the recessive version of the T-statistic. The distribution of the dominant version of the T-statistics for recessive gene is:
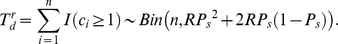



The distribution of the additive version of the T-statistic 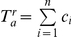 follows an extension of the binomial distribution, which we call the “trinomial distribution”:
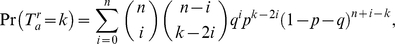
where 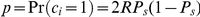, and 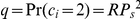. Throughout this work, we used this exact formula in our power calculations. As a note, this distribution can be approximated by a normal distribution when *n* is large, just like a binomial: 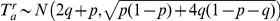.

For a gene with a dominant link, we assume that there is exactly one mutation on our gene of interest. For a patient, there is a probability *R* that the gene carries this mutation. When the gene carries the mutation, there is a probability 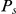 to discover it. Therefore, the distribution of the number of mutations under a dominant model would be 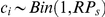, and the distribution of the additive version of the T-statistics and the distribution of the dominant version of the T-statistics are equal: 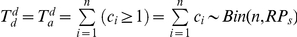. The recessive version of the T-statistics *Tr*, however, has zero power for detecting dominant variants.

The above discussions are focused on the contribution to the disease from single genes. In reality there could be *J* disease-causing genes 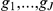, each 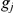 with a certain power 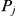 being identified by exome sequencing. As a result, the power of identifying any of them will be the 
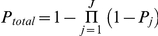
.

### Web Resources

Online Exome Power Calculator: http://exomepower.ssg.uab.edu

## Supporting Information

Figure S1 **The power difference of** ***Tr*** **-** ***Td*** **for recessive data for varying degrees of genetic heterogeneities (** ***R*** **-values) ranging from 0.01 to 1.** Other parameters are fixed to the default values: number of mutations *m* = 300; total number of genes *M* = 20,000; sensitivity of detecting mutations *Ps* = 0.8; and the mutation probability equals the genome-wide average *w* = 1.
(EPS)

Table S1
**The empirical type-I error rates of** ***Tr*** **,** ***Ta*** **, and** ***Td*** **by computer simulations.** Different combinations of sample sizes (*n*) and sensitivities of mutation detection (*Ps*) are explored. In each experiment *m* = 500 mutations are generated over *M* = 20,000 genes with the null distribution. The empirical type-I error is defined as the proportion of experiments when statistics *T* is greater than the cutoff determined by the Bonferroni-corrected 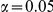 significant level (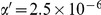), over the total of 1,000 experiments. It is clear that the type-I error rates are well-controlled in all cases (the small number of cases when the type-I error rates is greater than 0.05 are highlighted in bold), many are even too conservative due to the discrete nature of the binomial test.(DOC)

Table S2 **The calculated power of** ***Tr*** **for detecting a recessive gene with varying degrees of genetic heterogeneities (** ***R*** **) ranging from 0.01 to 1.** Other parameters are fixed to the default values: number of mutations *m* = 300; total number of genes *M* = 20,000; sensitivity of detecting mutations *Ps* = 0.8; and the mutation probability equals the genome-wide average (*w* = 1).
(DOC)

Table S3 **The power difference of** ***Tr*** **-** ***Ta*** **for recessive data for varying degrees of genetic heterogeneities (** ***R*** **-values) ranging from 0.01 to 1.** Negative numbers are highlighted in bold. Other parameters are fixed to the default values: number of mutations *m* = 300; total number of genes *M* = 20,000; sensitivity of detecting mutations *Ps* = 0.8; and the mutation probability equals the genome-wide average *w* = 1.
(DOC)

Table S4 **The power of** ***Td*** **for dominant data for varying degrees of genetic heterogeneities ranging from 0.01 to 1.** Other parameters are fixed to the default values: number of mutations *m* = 300; total number of genes *M* = 20,000; sensitivity of detecting mutations *Ps* = 0.8; and the mutation probability equals the genome-wide average *w* = 1.
(DOC)

Table S5 **The power of** ***Tr*** **for recessive data for varying degrees of sensitivities of mutation detection, ranging from 0.1 to 1.** Other parameters are fixed to the default values: number of mutations *m* = 300; total number of genes *M* = 20,000; genetic heterogeneity *R* = 0.05; and the mutation probability equals the genome-wide average *w* = 1.
(DOC)

Table S6 **The power of** ***Td*** **for dominant data for varying degrees of sensitivities of mutation detection, ranging from 0.1 to 1.** Other parameters are fixed to the default values: number of mutations *m* = 300; total number of genes *M* = 20,000; genetic heterogeneity *R* = 0.05; and the mutation probability equals the genome-wide average *w* = 1.
(DOC)

Table S7 **The power of** ***Tr*** **for recessive data for varying degrees of filtering efficiencies, ranging from 5 to 500.** Other parameters are fixed to the default values: genetic heterogeneity *R* = 0.05; total number of genes *M* = 20,000; sensitivity of detecting mutations *Ps* = 0.8; and the mutation probability equals the genome-wide average *w* = 1.
(DOC)

Table S8 **The power of** ***Td*** **for dominant data for varying degrees of filtering efficiencies, ranging from 5 to 500.** Other parameters are fixed to the default values: genetic heterogeneity *R* = 0.05; total number of genes *M* = 20,000; sensitivity of detecting mutations *Ps* = 0.8; and the mutation probability equals the genome-wide average *w* = 1.
(DOC)

Table S9 **The power of** ***Tr*** **for recessive data for varying degrees of relative mutation probabilities, ranging from 0.1 to 10 times of the genome average.** Other parameters are fixed to the default values: number of mutations *m* = 300; genetic heterogeneity *R* = 0.05; total number of genes *M* = 20,000; and sensitivity of detecting mutations *Ps* = 0.8.
(DOC)

Table S10 **The power of** ***Td*** **for dominant data for varying degrees of relative mutation probabilities, ranging from 0.1 to 10 times of the genome average.** Other parameters are fixed to the default values: number of mutations *m* = 300; genetic heterogeneity *R* = 0.05; total number of genes *M* = 20,000; and sensitivity of detecting mutations *Ps* = 0.8; and the filtering efficiency m = 300.
(DOC)

Document S1 **Proofs of claims.**
(DOC)
