# Supplementary material for: Statistical Guidance for Experimental Design and Data Analysis of Mutation Detection in Rare Monogenic Mendelian Diseases by Exome Sequencing
Source: PLoS One. 2012 Feb 10;7(2):e31358. doi: 10.1371/journal.pone.0031358 (PMC3277495; doi:10.1371/journal.pone.0031358)
Supplement: Table S1 — The empirical type-I error rates of Tr , Ta , and Td by computer simulations. Different combinations of sample sizes (n) and sensitivities of mutation detection (Ps) are explored. In each experiment m = 500 mutations are generated over M = 20,000 genes with the null distribution. The empirical type-I error is defined as the proportion of experiments when statistics T is greater than the cutoff determined by the Bonferroni-corrected significant level (), over the total of 1,000 experiments. It is clear that the type-I error rates are well-controlled in all cases (the small number of cases when the type-I error rates is greater than 0.05 are highlighted in bold), many are even too conservative due to the discrete nature of the binomial test. (DOC) [file pone.0031358.s002.doc]

| Statistics | *n* | *Ps* | | | | | | | | | |
| --- | --- | --- | --- | --- | --- | --- | --- | --- | --- | --- | --- |
|  |  | 0.1 | 0.2 | 0.3 | 0.4 | 0.5 | 0.6 | 0.7 | 0.8 | 0.9 | 1 |
| *Tr* | 2 | 0.002 | 0.007 | 0.02 | 0 | 0 | 0 | 0 | 0 | 0.001 | 0 |
|  | 5 | 0.003 | 0.017 | 0 | 0 | 0 | 0 | 0 | 0.002 | 0.012 | 0 |
|  | 10 | 0.004 | 0.029 | 0 | 0 | 0 | 0 | 0.001 | 0.007 | 0 | 0 |
|  | 20 | 0.008 | 0 | 0 | 0 | 0 | 0.001 | 0.012 | 0 | 0 | 0.001 |
|  | 30 | 0.014 | 0 | 0 | 0 | 0 | 0.003 | 0 | 0 | 0 | 0.002 |
|  | 40 | 0.02 | 0 | 0 | 0 | 0 | 0.003 | 0 | 0 | 0.001 | 0.004 |
|  | 50 | 0.026 | 0 | 0 | 0 | 0 | 0.005 | 0 | 0 | 0.001 | 0 |
|  | 60 | 0 | 0 | 0 | 0 | 0 | 0.006 | 0 | 0 | 0.004 | 0.001 |
|  | 70 | 0 | 0 | 0 | 0 | 0 | 0.006 | 0 | 0.003 | 0 | 0.001 |
|  | 80 | 0 | 0 | 0 | 0 | 0 | 0.006 | 0 | 0.004 | 0 | 0.001 |
|  | 90 | 0 | 0 | 0 | 0 | 0 | 0.009 | 0 | 0.004 | 0 | 0.001 |
|  | 100 | 0 | 0 | 0 | 0 | 0 | 0 | 0.001 | 0.004 | 0 | 0.001 |
| *Ta* | 2 | 0.004 | 0.014 | 0.037 | 0 | 0.001 | 0.008 | 0.022 | 0.001 | 0.001 | 0.001 |
|  | 5 | 0.018 | 0 | 0 | 0.001 | 0.011 | 0 | 0.004 | 0.026 | 0.002 | 0.004 |
|  | 10 | 0 | 0 | 0.002 | 0.012 | 0 | 0.005 | 0.003 | 0.013 | 0.043 | 0.006 |
|  | 20 | 0 | 0.004 | 0.03 | 0.002 | 0.004 | 0.003 | **0.053** | 0.019 | 0.002 | 0.023 |
|  | 30 | 0 | 0.017 | 0.002 | 0.003 | 0.031 | 0.016 | 0.017 | 0.01 | 0.005 | 0.022 |
|  | 40 | 0.001 | 0.029 | 0.003 | 0.019 | 0.001 | 0.044 | 0 | 0.007 | **0.053** | 0.019 |
|  | 50 | 0.003 | 0.001 | 0.004 | 0.035 | 0.006 | 0.004 | 0.014 | 0.026 | 0.024 | 0.015 |
|  | 60 | 0.01 | 0.002 | 0.008 | 0.003 | 0.012 | 0.011 | 0.009 | 0.01 | 0.017 | 0.008 |
|  | 70 | 0.016 | 0.004 | 0.019 | 0.004 | 0.026 | 0.027 | 0.019 | 0.037 | 0.048 | 0.044 |
|  | 80 | 0.024 | 0.004 | 0.033 | 0.007 | 0.044 | 0.008 | 0.042 | 0.011 | 0.021 | 0.02 |
|  | 90 | 0.038 | 0.005 | 0.002 | 0.01 | 0.003 | 0.013 | 0.011 | 0.034 | 0.012 | 0.017 |
|  | 100 | 0 | 0.008 | 0.004 | 0.018 | 0.005 | 0.018 | 0.026 | 0.014 | 0.03 | **0.057** |
| *Td* | 2 | 0.002 | 0.007 | 0.017 | 0.039 | 0 | 0 | 0 | 0 | 0 | 0 |
|  | 5 | 0.015 | 0.041 | 0 | 0.001 | 0.004 | 0.027 | 0.001 | 0.006 | 0.018 | 0.035 |
|  | 10 | 0 | 0 | 0.002 | 0.01 | 0.038 | 0.004 | 0.047 | 0.002 | 0.012 | **0.056** |
|  | 20 | 0 | 0.004 | 0.029 | 0.002 | 0.003 | 0.002 | 0.032 | 0.007 | 0.039 | 0.011 |
|  | 30 | 0 | 0.015 | 0.002 | 0.003 | 0.025 | 0.012 | 0.013 | 0.004 | 0.038 | 0.008 |
|  | 40 | 0.001 | 0.025 | 0.003 | 0.016 | 0.001 | 0.039 | 0 | 0.05 | 0.023 | 0.007 |
|  | 50 | 0.003 | **0.051** | 0.004 | 0.032 | 0.004 | 0.004 | 0.012 | 0.019 | 0.015 | 0.006 |
|  | 60 | 0.01 | 0.002 | 0.008 | **0.066** | 0.009 | 0.01 | **0.061** | 0.007 | 0.009 | 0.038 |
|  | 70 | 0.016 | 0.004 | 0.019 | 0.004 | 0.021 | 0.025 | 0.014 | 0.021 | 0.025 | 0.018 |
|  | 80 | 0.024 | 0.004 | 0.032 | 0.007 | 0.04 | 0.006 | 0.031 | 0.006 | 0.012 | 0.009 |
|  | 90 | 0.038 | 0.005 | **0.054** | 0.01 | 0.002 | 0.009 | 0.007 | 0.02 | 0.047 | 0.008 |
|  | 100 | 0.048 | 0.008 | 0.004 | 0.016 | 0.004 | 0.015 | 0.02 | 0.008 | 0.014 | 0.026 |
