# Supplementary material for: Statistical Guidance for Experimental Design and Data Analysis of Mutation Detection in Rare Monogenic Mendelian Diseases by Exome Sequencing
Source: PLoS One. 2012 Feb 10;7(2):e31358. doi: 10.1371/journal.pone.0031358 (PMC3277495; doi:10.1371/journal.pone.0031358)
Supplement: Table S2 — The calculated power of Tr for detecting a recessive gene with varying degrees of genetic heterogeneities ( R ) ranging from 0.01 to 1. Other parameters are fixed to the default values: number of mutations m = 300; total number of genes M = 20,000; sensitivity of detecting mutations Ps = 0.8; and the mutation probability equals the genome-wide average (w = 1). (DOC) [file pone.0031358.s003.doc]

| *n* | *R* | | | | | | | | | | | | | | | | | | | |
| --- | --- | --- | --- | --- | --- | --- | --- | --- | --- | --- | --- | --- | --- | --- | --- | --- | --- | --- | --- | --- |
|  | 0.01 | 0.02 | 0.03 | 0.04 | 0.05 | 0.06 | 0.07 | 0.08 | 0.09 | 0.10 | 0.15 | 0.20 | 0.30 | 0.40 | 0.50 | 0.60 | 0.70 | 0.80 | 0.90 | 1.00 |
| 1 | 0.00 | 0.00 | 0.00 | 0.00 | 0.00 | 0.00 | 0.00 | 0.00 | 0.00 | 0.00 | 0.00 | 0.00 | 0.00 | 0.00 | 0.00 | 0.00 | 0.00 | 0.00 | 0.00 | 0.00 |
| 2 | 0.00 | 0.00 | 0.00 | 0.00 | 0.00 | 0.00 | 0.00 | 0.00 | 0.00 | 0.00 | 0.01 | 0.02 | 0.04 | 0.07 | 0.10 | 0.15 | 0.20 | 0.26 | 0.33 | 0.41 |
| 5 | 0.00 | 0.00 | 0.00 | 0.01 | 0.01 | 0.01 | 0.02 | 0.02 | 0.03 | 0.04 | 0.08 | 0.13 | 0.25 | 0.38 | 0.51 | 0.63 | 0.74 | 0.83 | 0.89 | 0.94 |
| 10 | 0.00 | 0.01 | 0.01 | 0.03 | 0.04 | 0.05 | 0.07 | 0.09 | 0.11 | 0.13 | 0.25 | 0.37 | 0.60 | 0.77 | 0.88 | 0.94 | 0.98 | 0.99 | 1.00 | 1.00 |
| 20 | 0.00 | 0.00 | 0.01 | 0.01 | 0.02 | 0.04 | 0.06 | 0.08 | 0.10 | 0.13 | 0.30 | 0.48 | 0.77 | 0.92 | 0.98 | 0.99 | 1.00 | 1.00 | 1.00 | 1.00 |
| 50 | 0.00 | 0.03 | 0.07 | 0.14 | 0.21 | 0.30 | 0.39 | 0.48 | 0.56 | 0.63 | 0.87 | 0.96 | 1.00 | 1.00 | 1.00 | 1.00 | 1.00 | 1.00 | 1.00 | 1.00 |
| 100 | 0.03 | 0.14 | 0.30 | 0.47 | 0.62 | 0.74 | 0.83 | 0.89 | 0.93 | 0.96 | 1.00 | 1.00 | 1.00 | 1.00 | 1.00 | 1.00 | 1.00 | 1.00 | 1.00 | 1.00 |
| 200 | 0.04 | 0.25 | 0.54 | 0.76 | 0.89 | 0.95 | 0.98 | 0.99 | 1.00 | 1.00 | 1.00 | 1.00 | 1.00 | 1.00 | 1.00 | 1.00 | 1.00 | 1.00 | 1.00 | 1.00 |
| 500 | 0.22 | 0.77 | 0.96 | 1.00 | 1.00 | 1.00 | 1.00 | 1.00 | 1.00 | 1.00 | 1.00 | 1.00 | 1.00 | 1.00 | 1.00 | 1.00 | 1.00 | 1.00 | 1.00 | 1.00 |
| 1000 | 0.62 | 0.99 | 1.00 | 1.00 | 1.00 | 1.00 | 1.00 | 1.00 | 1.00 | 1.00 | 1.00 | 1.00 | 1.00 | 1.00 | 1.00 | 1.00 | 1.00 | 1.00 | 1.00 | 1.00 |
