# Supplementary material for: Statistical Guidance for Experimental Design and Data Analysis of Mutation Detection in Rare Monogenic Mendelian Diseases by Exome Sequencing
Source: PLoS One. 2012 Feb 10;7(2):e31358. doi: 10.1371/journal.pone.0031358 (PMC3277495; doi:10.1371/journal.pone.0031358)
Supplement: Table S3 — The power difference of Tr - Ta for recessive data for varying degrees of genetic heterogeneities ( R -values) ranging from 0.01 to 1. Negative numbers are highlighted in bold. Other parameters are fixed to the default values: number of mutations m = 300; total number of genes M = 20,000; sensitivity of detecting mutations Ps = 0.8; and the mutation probability equals the genome-wide average w = 1. (DOC) [file pone.0031358.s004.doc]

| *n* | *R* | | | | | | | | | | | | | | | | | | | |
| --- | --- | --- | --- | --- | --- | --- | --- | --- | --- | --- | --- | --- | --- | --- | --- | --- | --- | --- | --- | --- |
|  | 0.01 | 0.02 | 0.03 | 0.04 | 0.05 | 0.06 | 0.07 | 0.08 | 0.09 | 0.10 | 0.15 | 0.20 | 0.30 | 0.40 | 0.50 | 0.60 | 0.70 | 0.80 | 0.90 | 1.00 |
| 1 | 0.00 | 0.00 | 0.00 | 0.00 | 0.00 | 0.00 | 0.00 | 0.00 | 0.00 | 0.00 | 0.00 | 0.00 | 0.00 | 0.00 | 0.00 | 0.00 | 0.00 | 0.00 | 0.00 | 0.00 |
| 2 | 0.00 | 0.00 | 0.00 | 0.00 | 0.00 | 0.00 | 0.00 | 0.00 | 0.00 | 0.00 | 0.00 | 0.00 | 0.00 | 0.00 | 0.00 | 0.00 | 0.00 | 0.00 | 0.00 | 0.00 |
| 5 | 0.00 | 0.00 | 0.00 | 0.00 | 0.00 | 0.00 | 0.00 | 0.00 | 0.00 | 0.00 | **0.01** | **0.01** | **0.03** | **0.06** | **0.08** | **0.11** | **0.12** | **0.11** | **0.09** | **0.06** |
| 10 | 0.00 | 0.01 | 0.01 | 0.02 | 0.03 | 0.04 | 0.05 | 0.06 | 0.07 | 0.08 | 0.12 | 0.12 | 0.09 | 0.03 | **0.01** | **0.02** | **0.02** | **0.01** | 0.00 | 0.00 |
| 20 | 0.00 | 0.00 | 0.00 | 0.00 | 0.00 | **0.01** | **0.01** | **0.02** | **0.02** | **0.03** | **0.08** | **0.11** | **0.11** | **0.06** | **0.02** | **0.01** | 0.00 | 0.00 | 0.00 | 0.00 |
| 50 | 0.00 | 0.02 | 0.05 | 0.08 | 0.11 | 0.13 | 0.14 | 0.13 | 0.12 | 0.10 | 0.02 | **0.01** | 0.00 | 0.00 | 0.00 | 0.00 | 0.00 | 0.00 | 0.00 | 0.00 |
| 100 | 0.03 | 0.13 | 0.26 | 0.36 | 0.39 | 0.36 | 0.29 | 0.22 | 0.15 | 0.10 | 0.01 | 0.00 | 0.00 | 0.00 | 0.00 | 0.00 | 0.00 | 0.00 | 0.00 | 0.00 |
| 200 | 0.04 | 0.24 | 0.42 | 0.42 | 0.29 | 0.16 | 0.07 | 0.03 | 0.01 | 0.00 | 0.00 | 0.00 | 0.00 | 0.00 | 0.00 | 0.00 | 0.00 | 0.00 | 0.00 | 0.00 |
| 500 | 0.22 | 0.68 | 0.45 | 0.12 | 0.02 | 0.00 | 0.00 | 0.00 | 0.00 | 0.00 | 0.00 | 0.00 | 0.00 | 0.00 | 0.00 | 0.00 | 0.00 | 0.00 | 0.00 | 0.00 |
| 1000 | 0.62 | 0.72 | 0.10 | 0.00 | 0.00 | 0.00 | 0.00 | 0.00 | 0.00 | 0.00 | 0.00 | 0.00 | 0.00 | 0.00 | 0.00 | 0.00 | 0.00 | 0.00 | 0.00 | 0.00 |
