# Supplementary material for: Statistical Guidance for Experimental Design and Data Analysis of Mutation Detection in Rare Monogenic Mendelian Diseases by Exome Sequencing
Source: PLoS One. 2012 Feb 10;7(2):e31358. doi: 10.1371/journal.pone.0031358 (PMC3277495; doi:10.1371/journal.pone.0031358)
Supplement: Table S5 — The power of Tr for recessive data for varying degrees of sensitivities of mutation detection, ranging from 0.1 to 1. Other parameters are fixed to the default values: number of mutations m = 300; total number of genes M = 20,000; genetic heterogeneity R = 0.05; and the mutation probability equals the genome-wide average w = 1. (DOC) [file pone.0031358.s006.doc]

| *n* | *Ps* | | | | | | | | | |
| --- | --- | --- | --- | --- | --- | --- | --- | --- | --- | --- |
|  | 0.1 | 0.2 | 0.3 | 0.4 | 0.5 | 0.6 | 0.7 | 0.8 | 0.9 | 1 |
| 1 | 0 | 0 | 0 | 0 | 0 | 0 | 0 | 0 | 0 | 0 |
| 2 | 0.000 | 0.000 | 0.000 | 0.000 | 0.000 | 0.000 | 0.001 | 0.001 | 0.002 | 0.002 |
| 5 | 0.000 | 0.000 | 0.000 | 0.001 | 0.002 | 0.003 | 0.006 | 0.010 | 0.015 | 0.023 |
| 10 | 0.000 | 0.000 | 0.001 | 0.003 | 0.007 | 0.013 | 0.024 | 0.039 | 0.059 | 0.086 |
| 20 | 0.000 | 0.000 | 0.000 | 0.001 | 0.002 | 0.005 | 0.012 | 0.025 | 0.045 | 0.075 |
| 50 | 0.000 | 0.000 | 0.002 | 0.008 | 0.025 | 0.061 | 0.124 | 0.215 | 0.330 | 0.459 |
| 100 | 0.000 | 0.001 | 0.011 | 0.047 | 0.130 | 0.269 | 0.445 | 0.624 | 0.775 | 0.882 |
| 200 | 0.000 | 0.001 | 0.013 | 0.078 | 0.242 | 0.486 | 0.724 | 0.885 | 0.963 | 0.991 |
| 500 | 0.000 | 0.004 | 0.078 | 0.371 | 0.749 | 0.947 | 0.994 | 1.000 | 1.000 | 1.000 |
| 1000 | 0.000 | 0.016 | 0.297 | 0.810 | 0.986 | 1.000 | 1.000 | 1.000 | 1.000 | 1.000 |
