# Supplementary material for: Statistical Guidance for Experimental Design and Data Analysis of Mutation Detection in Rare Monogenic Mendelian Diseases by Exome Sequencing
Source: PLoS One. 2012 Feb 10;7(2):e31358. doi: 10.1371/journal.pone.0031358 (PMC3277495; doi:10.1371/journal.pone.0031358)
Supplement: Table S6 — The power of Td for dominant data for varying degrees of sensitivities of mutation detection, ranging from 0.1 to 1. Other parameters are fixed to the default values: number of mutations m = 300; total number of genes M = 20,000; genetic heterogeneity R = 0.05; and the mutation probability equals the genome-wide average w = 1. (DOC) [file pone.0031358.s007.doc]

| *n* | *Ps* | | | | | | | | | |
| --- | --- | --- | --- | --- | --- | --- | --- | --- | --- | --- |
|  | 0.1 | 0.2 | 0.3 | 0.4 | 0.5 | 0.6 | 0.7 | 0.8 | 0.9 | 1 |
| 1 | 0 | 0 | 0 | 0 | 0 | 0 | 0 | 0 | 0 | 0 |
| 2 | 0.000 | 0.000 | 0.000 | 0.000 | 0.000 | 0.000 | 0.000 | 0.000 | 0.000 | 0.000 |
| 5 | 0.000 | 0.000 | 0.000 | 0.000 | 0.000 | 0.000 | 0.000 | 0.000 | 0.000 | 0.000 |
| 10 | 0.000 | 0.000 | 0.000 | 0.000 | 0.000 | 0.000 | 0.000 | 0.000 | 0.000 | 0.000 |
| 20 | 0.000 | 0.000 | 0.000 | 0.000 | 0.000 | 0.000 | 0.000 | 0.000 | 0.000 | 0.000 |
| 50 | 0.000 | 0.000 | 0.000 | 0.000 | 0.000 | 0.000 | 0.000 | 0.001 | 0.002 | 0.003 |
| 100 | 0.000 | 0.000 | 0.000 | 0.000 | 0.000 | 0.000 | 0.001 | 0.002 | 0.005 | 0.011 |
| 200 | 0.000 | 0.000 | 0.000 | 0.000 | 0.000 | 0.001 | 0.005 | 0.015 | 0.038 | 0.078 |
| 500 | 0.000 | 0.000 | 0.000 | 0.000 | 0.002 | 0.018 | 0.077 | 0.208 | 0.403 | 0.610 |
| 1000 | 0.000 | 0.000 | 0.000 | 0.001 | 0.021 | 0.154 | 0.455 | 0.763 | 0.930 | 0.986 |
