# Supplementary material for: Statistical Guidance for Experimental Design and Data Analysis of Mutation Detection in Rare Monogenic Mendelian Diseases by Exome Sequencing
Source: PLoS One. 2012 Feb 10;7(2):e31358. doi: 10.1371/journal.pone.0031358 (PMC3277495; doi:10.1371/journal.pone.0031358)
Supplement: Table S7 — The power of Tr for recessive data for varying degrees of filtering efficiencies, ranging from 5 to 500. Other parameters are fixed to the default values: genetic heterogeneity R = 0.05; total number of genes M = 20,000; sensitivity of detecting mutations Ps = 0.8; and the mutation probability equals the genome-wide average w = 1. (DOC) [file pone.0031358.s008.doc]

| *n* | *m* | | | | | | | | | |
| --- | --- | --- | --- | --- | --- | --- | --- | --- | --- | --- |
|  | 5 | 10 | 20 | 30 | 50 | 100 | 200 | 300 | 400 | 500 |
| 1 | 0.032 | 0.032 | 0.032 | 0.032 | 0 | 0 | 0 | 0 | 0 | 0 |
| 2 | 0.063 | 0.063 | 0.063 | 0.001 | 0.001 | 0.001 | 0.001 | 0.001 | 0.001 | 0.001 |
| 5 | 0.150 | 0.150 | 0.010 | 0.010 | 0.010 | 0.010 | 0.010 | 0.010 | 0.010 | 0.000 |
| 10 | 0.278 | 0.278 | 0.039 | 0.039 | 0.039 | 0.039 | 0.039 | 0.039 | 0.003 | 0.003 |
| 20 | 0.478 | 0.133 | 0.133 | 0.133 | 0.133 | 0.133 | 0.133 | 0.025 | 0.025 | 0.025 |
| 50 | 0.803 | 0.478 | 0.478 | 0.478 | 0.478 | 0.478 | 0.215 | 0.215 | 0.215 | 0.076 |
| 100 | 0.833 | 0.833 | 0.833 | 0.833 | 0.833 | 0.624 | 0.624 | 0.624 | 0.398 | 0.398 |
| 200 | 0.989 | 0.989 | 0.989 | 0.989 | 0.989 | 0.956 | 0.956 | 0.885 | 0.885 | 0.769 |
| 500 | 1.000 | 1.000 | 1.000 | 1.000 | 1.000 | 1.000 | 1.000 | 1.000 | 1.000 | 0.999 |
| 1000 | 1.000 | 1.000 | 1.000 | 1.000 | 1.000 | 1.000 | 1.000 | 1.000 | 1.000 | 1.000 |
