# Supplementary material for: Statistical Guidance for Experimental Design and Data Analysis of Mutation Detection in Rare Monogenic Mendelian Diseases by Exome Sequencing
Source: PLoS One. 2012 Feb 10;7(2):e31358. doi: 10.1371/journal.pone.0031358 (PMC3277495; doi:10.1371/journal.pone.0031358)
Supplement: Table S8 — The power of Td for dominant data for varying degrees of filtering efficiencies, ranging from 5 to 500. Other parameters are fixed to the default values: genetic heterogeneity R = 0.05; total number of genes M = 20,000; sensitivity of detecting mutations Ps = 0.8; and the mutation probability equals the genome-wide average w = 1. (DOC) [file pone.0031358.s009.doc]

| *n* | *m* | | | | | | | | | |
| --- | --- | --- | --- | --- | --- | --- | --- | --- | --- | --- |
|  | 5 | 10 | 20 | 30 | 50 | 100 | 200 | 300 | 400 | 500 |
| 1 | 0 | 0 | 0 | 0 | 0 | 0 | 0 | 0 | 0 | 0 |
| 2 | 0.002 | 0.002 | 0.002 | 0.002 | 0.000 | 0.000 | 0.000 | 0.000 | 0.000 | 0.000 |
| 5 | 0.015 | 0.015 | 0.001 | 0.001 | 0.001 | 0.001 | 0.000 | 0.000 | 0.000 | 0.000 |
| 10 | 0.006 | 0.006 | 0.006 | 0.006 | 0.006 | 0.000 | 0.000 | 0.000 | 0.000 | 0.000 |
| 20 | 0.044 | 0.044 | 0.044 | 0.007 | 0.007 | 0.001 | 0.001 | 0.000 | 0.000 | 0.000 |
| 50 | 0.323 | 0.323 | 0.139 | 0.139 | 0.049 | 0.014 | 0.004 | 0.001 | 0.000 | 0.000 |
| 100 | 0.768 | 0.571 | 0.371 | 0.371 | 0.212 | 0.106 | 0.019 | 0.002 | 0.001 | 0.000 |
| 200 | 0.960 | 0.905 | 0.905 | 0.814 | 0.692 | 0.407 | 0.107 | 0.015 | 0.003 | 0.000 |
| 500 | 1.000 | 1.000 | 1.000 | 0.999 | 0.996 | 0.964 | 0.622 | 0.208 | 0.049 | 0.007 |
| 1000 | 1.000 | 1.000 | 1.000 | 1.000 | 1.000 | 1.000 | 0.982 | 0.763 | 0.280 | 0.049 |
