# Supplementary material for: Statistical Guidance for Experimental Design and Data Analysis of Mutation Detection in Rare Monogenic Mendelian Diseases by Exome Sequencing
Source: PLoS One. 2012 Feb 10;7(2):e31358. doi: 10.1371/journal.pone.0031358 (PMC3277495; doi:10.1371/journal.pone.0031358)
Supplement: Table S9 — The power of Tr for recessive data for varying degrees of relative mutation probabilities, ranging from 0.1 to 10 times of the genome average. Other parameters are fixed to the default values: number of mutations m = 300; genetic heterogeneity R = 0.05; total number of genes M = 20,000; and sensitivity of detecting mutations Ps = 0.8. (DOC) [file pone.0031358.s010.doc]

| *n* | *w* | | | | | | |
| --- | --- | --- | --- | --- | --- | --- | --- |
|  | 0.1 | 0.2 | 0.5 | 1 | 2 | 5 | 10 |
| 1 | 0 | 0 | 0 | 0 | 0 | 0 | 0 |
| 2 | 0.001 | 0.001 | 0.001 | 0.001 | 0.001 | 0.000 | 0.000 |
| 5 | 0.010 | 0.010 | 0.010 | 0.010 | 0.000 | 0.000 | 0.000 |
| 10 | 0.039 | 0.039 | 0.039 | 0.039 | 0.003 | 0.000 | 0.000 |
| 20 | 0.133 | 0.133 | 0.133 | 0.025 | 0.025 | 0.003 | 0.000 |
| 50 | 0.478 | 0.478 | 0.215 | 0.215 | 0.076 | 0.022 | 0.000 |
| 100 | 0.833 | 0.833 | 0.624 | 0.624 | 0.398 | 0.042 | 0.000 |
| 200 | 0.989 | 0.956 | 0.956 | 0.885 | 0.769 | 0.194 | 0.001 |
| 500 | 1.000 | 1.000 | 1.000 | 1.000 | 0.996 | 0.811 | 0.004 |
| 1000 | 1.000 | 1.000 | 1.000 | 1.000 | 1.000 | 0.995 | 0.016 |
