# Supplementary material for: Statistical Guidance for Experimental Design and Data Analysis of Mutation Detection in Rare Monogenic Mendelian Diseases by Exome Sequencing
Source: PLoS One. 2012 Feb 10;7(2):e31358. doi: 10.1371/journal.pone.0031358 (PMC3277495; doi:10.1371/journal.pone.0031358)
Supplement: Table S10 — The power of Td for dominant data for varying degrees of relative mutation probabilities, ranging from 0.1 to 10 times of the genome average. Other parameters are fixed to the default values: number of mutations m = 300; genetic heterogeneity R = 0.05; total number of genes M = 20,000; and sensitivity of detecting mutations Ps = 0.8; and the filtering efficiency m = 300. (DOC) [file pone.0031358.s011.doc]

| *n* | *w* | | | | | | |
| --- | --- | --- | --- | --- | --- | --- | --- |
|  | 0.1 | 0.2 | 0.5 | 1 | 2 | 5 | 10 |
| 1 | 0 | 0 | 0 | 0 | 0 | 0 | 0 |
| 2 | 0.000 | 0.000 | 0.000 | 0.000 | 0.000 | 0.000 | 0.000 |
| 5 | 0.001 | 0.001 | 0.000 | 0.000 | 0.000 | 0.000 | 0.000 |
| 10 | 0.000 | 0.000 | 0.000 | 0.000 | 0.000 | 0.000 | 0.000 |
| 20 | 0.007 | 0.007 | 0.001 | 0.000 | 0.000 | 0.000 | 0.000 |
| 50 | 0.049 | 0.049 | 0.004 | 0.001 | 0.000 | 0.000 | 0.000 |
| 100 | 0.212 | 0.106 | 0.048 | 0.002 | 0.000 | 0.000 | 0.000 |
| 200 | 0.692 | 0.550 | 0.180 | 0.015 | 0.000 | 0.000 | 0.000 |
| 500 | 0.998 | 0.980 | 0.784 | 0.208 | 0.001 | 0.000 | 0.000 |
| 1000 | 1.000 | 1.000 | 0.998 | 0.763 | 0.004 | 0.000 | 0.000 |
