# Supplementary material for: Statistical Guidance for Experimental Design and Data Analysis of Mutation Detection in Rare Monogenic Mendelian Diseases by Exome Sequencing
Source: PLoS One. 2012 Feb 10;7(2):e31358. doi: 10.1371/journal.pone.0031358 (PMC3277495; doi:10.1371/journal.pone.0031358)
Supplement: Document S1 — Proofs of claims. (DOC) [file pone.0031358.s012.doc]

# Supporting Document S1: Proofs of claims 1 and 2.

**Claim 1: If and , then approximately .**

**Proof**:

.

The parenthesis contains m terms, all between and 1. Therefore,

,

or

.

This approximation bounds are quite tight as when *M*=20,000 and *m*=300.

**Claim 2: If and , then approximately .**

**Proof:**

Based on Claim 1,
